# Favipiravir elicits antiviral mutagenesis during virus replication in vivo

**DOI:** 10.7554/eLife.03679

**Published:** 2014-10-21

**Authors:** Armando Arias, Lucy Thorne, Ian Goodfellow

**Affiliations:** 1 Division of Virology, Addenbrooke's Hospital, University of Cambridge, Cambridge, United Kingdom; University of Utah, United States

**Keywords:** quasispecies, lethal mutagenesis, error-catastrophe, norovirus, favipiravir, polymerase fidelity, viruses

## Abstract

Lethal mutagenesis has emerged as a novel potential therapeutic approach to treat viral infections. Several studies have demonstrated that increases in the high mutation rates inherent to RNA viruses lead to viral extinction in cell culture, but evidence during infections in vivo is limited. In this study, we show that the broad-range antiviral nucleoside favipiravir reduces viral load in vivo by exerting antiviral mutagenesis in a mouse model for norovirus infection. Increased mutation frequencies were observed in samples from treated mice and were accompanied with lower or in some cases undetectable levels of infectious virus in faeces and tissues. Viral RNA isolated from treated animals showed reduced infectivity, a feature of populations approaching extinction during antiviral mutagenesis. These results suggest that favipiravir can induce norovirus mutagenesis in vivo, which in some cases leads to virus extinction, providing a proof-of-principle for the use of favipiravir derivatives or mutagenic nucleosides in the clinical treatment of noroviruses.

**DOI:** http://dx.doi.org/10.7554/eLife.03679.001

## eLife digest

Viruses can infect, take control of and replicate themselves inside the living cells of other organisms. Some viral diseases can be treated with antiviral drugs, which stop viral infections either by making it more difficult for viruses to enter cells or by preventing the virus replicating once inside. As antiviral drugs are currently only available to treat a handful of viral infections, efforts are underway to develop and test experimental antiviral drugs.

One such experimental drug is called favipiravir, which is proving to be effective against several viruses that store their genetic information in the form of RNA molecules. These viruses include those that cause diseases such as influenza, gastroenteritis, and Ebola. Along with ongoing work determining how safe and effective favipiravir is for treating viral infections, researchers are also attempting to better understand how favipiravir works.

Whenever a strand of RNA is copied to allow a new virus to form, there is a risk that mistakes—or mutations—that could harm the virus are introduced into the genetic code. Previous experiments performed on cells grown in the laboratory suggested that favipiravir works against RNA viruses by increasing how often these mutations occur. RNA viruses naturally experience a large number of mutations and the ability to make mutations is in fact a benefit for viruses as it allows them to evolve rapidly and to escape immune responses. However, there is a limit to how many mutations can be tolerated in the viral genome before it can no longer replicate. Therefore, a slight increase in how often mutations occur—as thought to be caused by favipiravir—is able to stop the RNA virus replicating and halt the infection. However, favipiravir's mode of action had yet to be confirmed in living animals.

Using mice, Arias et al. tested favipiravir's ability to treat a persistent infection by norovirus—the most common cause of viral gastroenteritis in humans and also responsible for life-threatening chronic diarrhoea in immunodeficient patients. Treatment increased the number of mutations that occurred when the viral RNA replicated and could reduce the amount of virus in the mice to undetectable levels. In addition, favipiravir did not show toxicity in mice after 8 weeks of treatment. This suggests that favipiravir has the potential to be used safely and effectively to treat norovirus and other RNA viruses, although further studies are required before it can be developed into a clinical treatment.

**DOI:** http://dx.doi.org/10.7554/eLife.03679.002

## Introduction

Due to elevated error frequencies during the replication of their genetic material, RNA virus populations exist as complex distributions of mutant genomes also known as quasispecies (Domingo et al., 2012). Genetic variability confers viral populations the flexibility to rapidly adapt to the environment, typically the host, and respond to different selective constraints such as the immune system or antiviral compounds (Domingo et al., 2008). As a consequence, changes in the replication fidelity of a virus can affect its virulence and transmission during natural infections (Pfeiffer and Kirkegaard, 2005; Vignuzzi et al., 2006; Bull et al., 2010). Recent evidence suggests that RNA virus replication error rates are finely balanced to generate ample diversity while maintaining sufficient accuracy in the transmission of genetic information (Pfeiffer and Kirkegaard, 2005; Vignuzzi et al., 2006; Levi et al., 2010; Gnädig et al., 2012; Sanz-Ramos et al., 2012; Rozen-Gagnon et al., 2014). Pioneering theoretical studies on self-replicating genomes suggested that any live organism has a maximum error rate tolerated to copy its genome. Given their elevated mutation rates, it was anticipated that RNA viruses exist close to their corresponding tolerated threshold (Swetina and Schuster, 1982; Domingo, 2000; Eigen, 2002). Hence, slight increases in virus mutation frequencies might result in the extinction of the replicating population (Eigen, 2002; Domingo et al., 2010). These predictions led to the proposal of lethal mutagenesis of viruses as a new therapeutic approach based on reducing the fidelity of genome replication.

Several nucleoside analogues (i.e., ribavirin, 5-fluorouracil, 5-azacytidine) and non-nucleoside compounds (amiloride) display antiviral activities in cell culture against a wide range of RNA viruses and appear to act via increased mutagenesis (Loeb et al., 1999; Crotty et al., 2000; Sierra et al., 2000; Grande-Pérez et al., 2002; Dapp et al., 2009). Increased mutation frequencies are accompanied with decreased virus progeny, infectivity and fitness, leading in some cases to the complete extinction of the virus population (Domingo et al., 2012).

Despite all the evidence in cell culture, data confirming lethal mutagenesis as a plausible therapeutic approach in vivo remains limited. Although some of the antiviral compounds with mutagenic activity in cell culture are also reported to have antiviral activity in vivo, controversy exists regarding the therapeutic mechanism. To date, the most successful antiviral compound with mutagenic activity in cell culture is purine analogue ribavirin (Crotty et al., 2000; Maag et al., 2001; Brochot et al., 2007; Perales et al., 2009; Moreno et al., 2011). Ribavirin is commonly used in combination with pegylated interferon in the treatment of hepatitis C virus (HCV) infections. There are contradictory results on whether the mode of action of ribavirin on HCV in vivo is due to mutagenesis, immunomodulatory activities, or other antiviral mechanisms (Graci and Cameron, 2006; Chevaliez et al., 2007; Lutchman et al., 2007; Perelson and Layden, 2007; Chung et al., 2013; Dietz et al., 2013). Although increased HCV mutation frequencies have been reported in several cases (Asahina et al., 2005; Dietz et al., 2013), some studies have not found increased mutagenesis as a factor contributing to the associated antiviral activity (Chevaliez et al., 2007; Lutchman et al., 2007). Therefore there remains an open question as to whether or not ribavirin-mediated antiviral activity in vivo is as a result of mutagenesis or one of the other reported mechanisms (Graci and Cameron, 2006; Chevaliez et al., 2007). Hence, further investigations are needed to demonstrate that lethal mutagenesis is a conceivable approach for the general treatment of RNA virus infections in vivo.

Several novel compounds eliciting antiviral mutagenesis in cell culture were recently identified (Levi et al., 2010; Mullins et al., 2011; Baranovich et al., 2013; Dapp et al., 2014). Among them, favipiravir is a novel broad spectrum nucleoside analogue which is effective in the control of a vast number of RNA viruses in vivo (Gowen et al., 2007; Furuta et al., 2009; Mendenhall et al., 2011; Baranovich et al., 2013; Caroline et al., 2014; Oestereich et al., 2014; Smither et al., 2014), although its therapeutic mechanism of action is still under study. Favipiravir was initially identified as an antiviral compound for the treatment of influenza virus infection (Furuta et al., 2002; Sidwell et al., 2007) whose activity correlates with increased mutagenesis in cell culture (Baranovich et al., 2013). A recent study demonstrated that favipiravir-triphosphate can be used as a substrate by the virus polymerase and incorporated ambiguously into RNA opposite C and U in the template molecule (Jin et al., 2013).

With the aim of demonstrating lethal mutagenesis as a conceivable approach to treat viral infections in vivo, we investigated whether ribavirin and favipiravir elicit antiviral activities in a mouse model of persistent norovirus infection. Favipiravir caused significant decreases in virus titres and viral RNA levels and led to the clearance of infectious virus in the faeces of seven out of nine animals (only two out of ten animals did not shed detectable infectious virus in control group). Blind passaging of faecal and tissue samples in cell culture confirmed that favipiravir treatment led to viral extinction in some animals. In contrast, ribavirin showed limited efficacy in vivo which correlated with lower antiviral activity in cell culture when compared to favipiravir. Favipiravir antiviral activity in vivo was associated with a significant increase in viral mutation frequencies. Viral RNA isolated from faeces of treated animals showed decreased specific infectivity, and infectious virus from faeces showed decreased fitness in tissue culture. These data suggest that favipiravir causes increased mutagenesis leading to decreased infectivity and fitness of viral genomes, both features of viral populations approaching extinction during mutagenesis. These results constitute a proof of concept for lethal mutagenesis in vivo and support antiviral therapies based on mutagenic compounds at the clinical level. The data also support the use of favipiravir in the treatment of norovirus infections for which there are not yet licenced antivirals or vaccines available and highlight a need for further studies on favipiravir and other improved derivatives as possible broad-range antiviral strategies.

## Results

### Ribavirin and favipiravir inhibit norovirus replication in cell culture

To investigate whether ribavirin and favipiravir elicit any antiviral activity on norovirus replication, we employed two different murine norovirus (MNV) strains, MNV-1 and MNV-3. MNV-1 was the first MNV strain isolated as the causative agent of a fatal infection in Stat1^−/−^ mice (Karst et al., 2003). Later studies identified many other MNV strains, including MNV-3, wide spread in different mice laboratory colonies (Hsu et al., 2006; Thackray et al., 2007; Barron et al., 2011). MNV is used as a model to study norovirus replication and pathogenesis and has facilitated a better understanding of the norovirus life cycle (Wobus et al., 2006). MNV-1 efficiently replicates in cell culture although it shows limited virulence in wild-type mice. In contrast, MNV-3 typically produces lower yields in tissue culture, yet establishes long-term persistent infections in wild-type mice, being detected for at least 8 months after inoculation (Arias et al., 2012a; McFadden et al., 2013). Previous studies demonstrated that ribavirin and favipiravir elicit antiviral activity against MNV-1 and human norovirus (HuNoV) replicon in cell culture (Chang and George, 2007; Rocha-Pereira et al., 2012). However, the mechanism of action and their utility in vivo have not been described.

**Figure 1.**
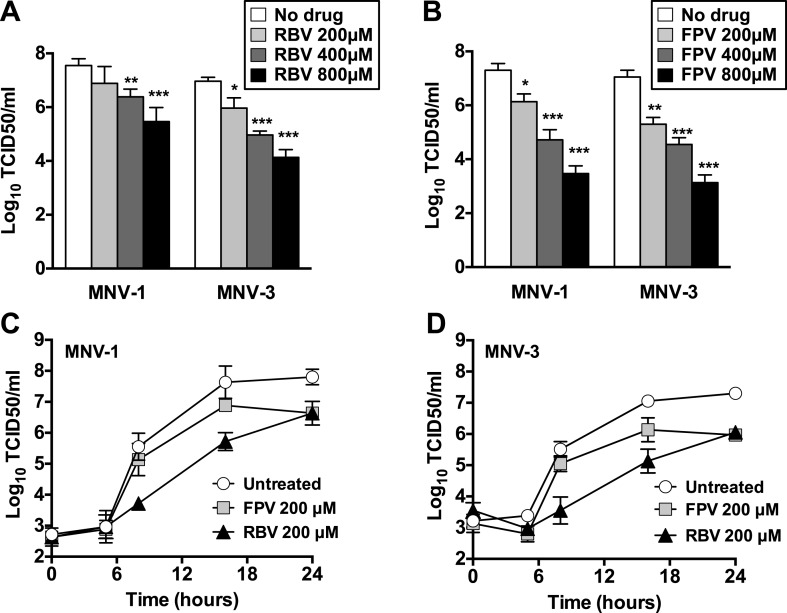
Ribavirin and favipiravir are efficient inhibitors of norovirus replication. (**A**) MNV-1 and MNV-3 viral yields obtained after infection of confluent monolayers of RAW264.7 cells in the absence (white bars) or presence of 200 (light grey), 400 (dark grey), or 800 μM ribavirin (black). MNV was inoculated at an MOI of 0.01 TCID50/cell and infections were allowed to proceed for 24 hr when cultures were freeze-thawed for virus release. (**B**) MNV-1 and MNV-3 viral yields obtained after infection of confluent monolayers of RAW264.7 cells in the absence (white bars) or presence of 200 (light grey), 400 (dark grey), or 800 μM favipiravir (black). MNV was inoculated at an MOI of 0.01 TCID50/cell and infections were allowed to proceed for 24 hr. Statistically significant differences are represented (p < 0.05, *; p < 0.01, **; p < 0.001, ***; 2-way ANOVA test). (**C** and **D**) Kinetics of MNV-1 and MNV-3 infection in the presence of ribavirin or favipiravir. Confluent monolayers of RAW264.7 cells were infected with MNV-1 (**C**) or MNV-3 (**D**) at an MOI of 5 TCID50/cell. Infected cell cultures were treated with 200 μM ribavirin (RBV) or favipiravir (FPV) as explained in ‘Materials and methods’. Replication kinetics of MNV-1 and MNV-3 in untreated infected cells are shown in parallel (DMEM). Every time point is the average of three biological replicates (±SD). **DOI:** http://dx.doi.org/10.7554/eLife.03679.003

The treatment of RAW264.7 cells infected with MNV (MOI of 0.01 TCID50/cell) with ribavirin or favipiravir resulted in significant reductions in virus yields, reaching up to a 3-log_10_ decrease for ribavirin and a 4-log_10_ for favipiravir (Figure 1). A decrease in cell viability was observed when cells were treated with high concentration of ribavirin, although this reduction never exceeded 50%. In contrast, no significant decrease in cell viability was observed in cells treated with favipiravir (Figure 1—figure supplement 1).

**Figure 1—figure supplement 1.**
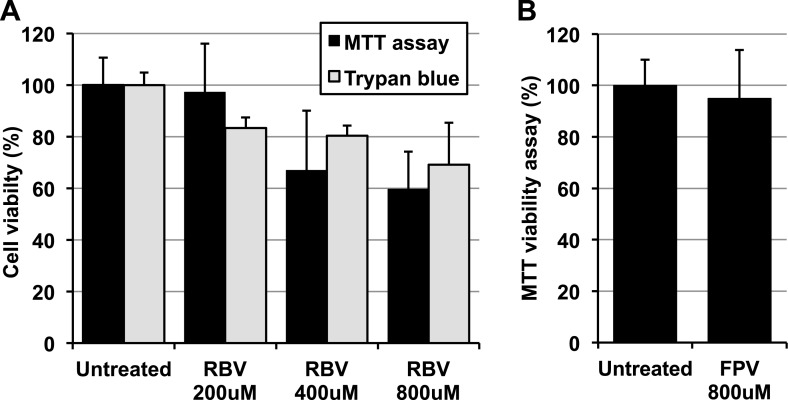
Favipiravir and ribavirin toxicity in RAW264.7 cells. (**A**) Ribavirin toxicity upon RAW264.7 cells was scored using both trypan blue, which measures the proportion of dead cells, and CellTiter-Blue Cell Viability Assay (Promega), which accounts for living active cells. (**B**) Favipiravir toxicity was determined by CellTiter-Blue Cell Viability Assay (Promega). **DOI:** http://dx.doi.org/10.7554/eLife.03679.004

To further investigate the mechanism of inhibition, we determined the effect of ribavirin and favipiravir upon MNV replication in single-cycle replication kinetics carried out by infecting RAW264.7 cells at high MOI. Favipiravir inhibition occurred in a gradual manner, with the greatest reduction in virus titre being observed at later time points (Figure 1C,D). This result is suggestive of a cumulative antiviral activity often observed with increasing number of mutations in viral genomes during successive rounds of replication. In contrast, ribavirin inhibited norovirus replication from an early time point (8 hr), suggesting a different mechanism of action, an additional antiviral activity relative to favipiravir, or potentially due to a different mutational spectrum activity elicited by these two compounds. However, it is alternatively possible that the delayed inhibition shown by favipiravir compared to ribavirin reflects the fact that it takes several more steps to convert favipiravir to the active form (Furuta et al., 2005). To investigate this possibility, we repeated the kinetics by incubating the cells overnight with favipiravir before infection to facilitate favipiravir conversion into favipiravir-triphosphate. No major differences were observed with cells pre-incubated during 1 hr or overnight with favipiravir (data not shown), which suggests that ribavirin might be a more potent inhibitor of viral RNA replication. To confirm this possibility, we determined the viral RNA synthesis kinetics for MNV-3 in the presence of both compounds and confirmed that ribavirin inhibits viral RNA synthesis from an early time post-infection, while the effects of favipiravir are only observed at later time points (data not shown).

### Ribavirin and favipiravir are mutagenic for MNV and cause decreased infectivity

**Figure 2.**
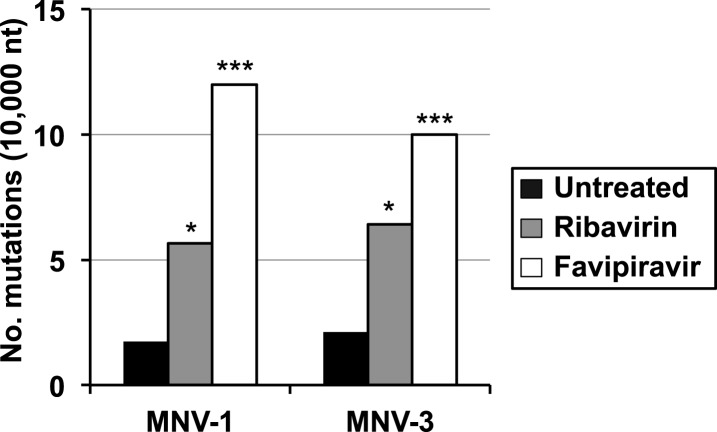
Increased mutation frequencies in virus quasispecies treated with ribavirin or favipiravir. Mutation frequencies are represented as the average number of mutations found every 10,000 nucleotides sequenced in MNV-1 and MNV-3 populations after 4 passages in RAW264.7 cells in the absence or presence of 200 μM ribavirin (RBV) or favipiravir (FPV) (p < 0.05, *; p < 0.001, ***, Mann–Whitney U test). **DOI:** http://dx.doi.org/10.7554/eLife.03679.005

Although ribavirin and favipiravir cause increased mutagenesis in several RNA viruses (Graci and Cameron, 2006; Moreno et al., 2011; Baranovich et al., 2013), the mechanism of antiviral activity against noroviruses is not known. To determine whether ribavirin and favipiravir treatment resulted in greater mutation frequencies, we carried out sequence analysis of individual molecular clones isolated from populations subjected to 4 passages in the presence of either 200 μM ribavirin or favipiravir. We found that both compounds caused significant increases in the mutation frequencies of replicating virus. Ribavirin treatment resulted in a ∼threefold increase while favipiravir led to a five to sixfold increase in the number of mutations per nucleotide (Figure 2). Importantly, alterations in the transition frequency patterns were also observed. Ribavirin treatment resulted in greater proportion of G to A and C to U transitions than in untreated virus populations, while favipiravir led to a slight increase in A to G and U to C transition rates (Table 1). These alterations are in agreement with those observed for other viruses treated with the same compounds (Agudo et al., 2010; Levi et al., 2010; Baranovich et al., 2013; Ortega-Prieto et al., 2013).

**Table 1.** Mutation type distribution in MNV populations treated with ribavirin and favipiravir **DOI:** http://dx.doi.org/10.7554/eLife.03679.006

	Untreated		Ribavirin		Favipiravir	
	MNV-1	MNV-3	MNV-1	MNV-3	MNV-1	MNV-3
A → G	4	4	1	3	9	5
U → C	1	3	1	2	3	7
G → A	0	2	5	2	2	0
C → U	4	1	4	4	0	4
Transversions	1	3	1	2	1	0
Deletions	1	0	1	0	0	0
Total nucleotides sequenced*	63,395	65,822	23,358	20,243	12,488	16,003

* Total number of nucleotides sequenced in each different untreated or treated population analysed.

Proportion of different types of mutations observed in untreated, or ribavirin- or favipiravir-treated MNV populations.

**Figure 3.**
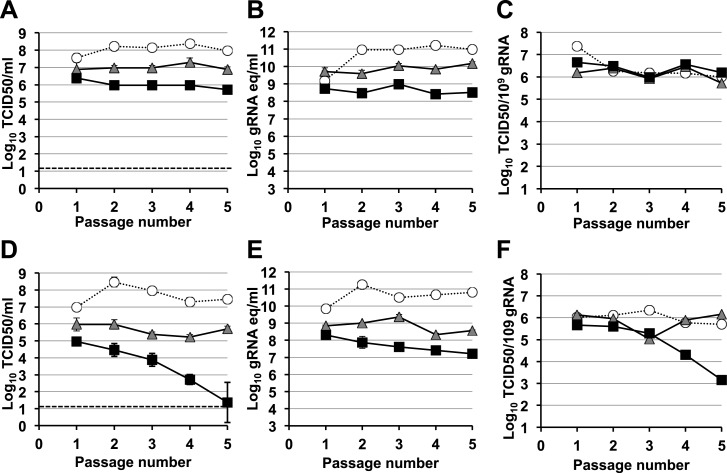
Murine norovirus titres decrease during serial passage in the presence of ribavirin. MNV-1 (**A**, **B**, **C**) and MNV-3 (**D**, **E**, **F**) were serially passaged in the absence (white circles) or presence of 200 (grey triangles) or 400 μM ribavirin (black squares). Virus was inoculated at an MOI of 0.1 TCID50/cell in passage 1. Subsequent passages were carried out with 200 μl (1/10 vol) of neat virus recovered from the previous passage. The different graphs show the resulting virus titres (**A** and **D**), genome copy equivalents (**B** and **E**), and the resulting specific infectivity determined for encapsidated genomes (**C** and **F**). Specific infectivity values were calculated as the number of infectious viruses (TCID50 units) found in 10^9^ genome copies from data obtained in **A**, **B**, **D**, and **E**. To calculate the number of genome copy equivalents, non-encapsidated genomes were removed before RNA extraction by micrococcal nuclease treatment. **DOI:** http://dx.doi.org/10.7554/eLife.03679.007

**Figure 4.**
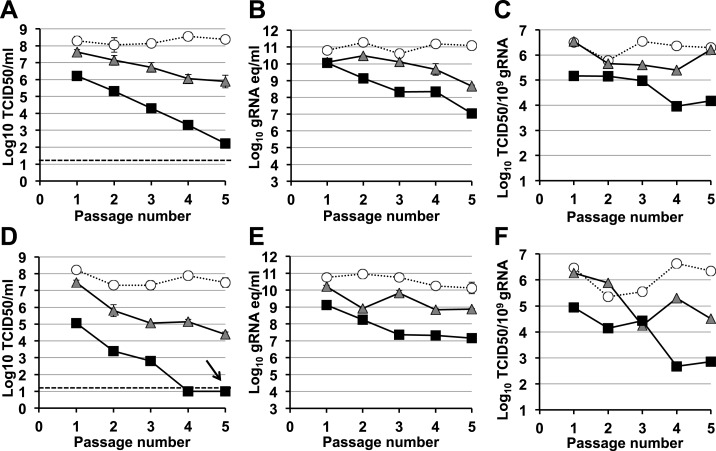
Murine norovirus infectivity decreases during serial passages in the presence of favipiravir. MNV-1 (**A**, **B**, **C**) and MNV-3 (**D**, **E**, **F**) were serially passaged in the absence (white circles), or presence of 200 (grey triangles) or 400 μM favipiravir (black squares). Passage 1 infections were carried out at an MOI of 0.1 TCID50/cell. Subsequent passages were carried out with 200 μl (1/10 vol) of neat virus recovered from the previous passage. The different graphs show the resulting virus titres (**A** and **D**), genome copy equivalents (**B** and **E**), and the resulting specific infectivity determined for encapsidated genomes (**C** and **F**). Specific infectivity values were calculated as the number of infectious viruses (TCID50 units) found in 10^9^ genome copies from data obtained in **A**, **B**, **D**, and **E**. To calculate the number of genome copy equivalents, non-encapsidated genomes were removed before RNA extraction by micrococcal nuclease treatment. The arrow in **D** indicates virus extinction confirmed by the absence of infectious virus and viral RNA (qPCR) after three serial passages in the absence of favipiravir. **DOI:** http://dx.doi.org/10.7554/eLife.03679.009

A signature feature for error catastrophe in virus populations subjected to mutagenesis is a decrease in virus specific infectivity (González-López et al., 2005; Grande-Pérez et al., 2005; Perales et al., 2009). To investigate whether these compounds reduced the infectivity of treated norovirus populations, we serially passaged MNV in the presence of either ribavirin or favipiravir (Figure 3, Figure 4, respectively). In both cases, lower virus titres and encapsidated viral RNA levels were observed during passage in cell culture. After 5 passages, virus titres for MNV-1 treated with 400 μM favipiravir, and MNV-3 treated with either 400 μM ribavirin or favipiravir were close to or below the detection limit, indicating that ribavirin and favipiravir can cause lethal mutagenesis of MNV. We confirmed that MNV-3 was extinguished after 5 passages in the presence of 400 μM favipiravir, by three consecutive blind passages in RAW264.7 cells in the absence of the drug. The specific infectivity, a measure of the ratio of infectious virus per encapsidated genome, was consistently reduced during passages in the presence of favipiravir for both MNV strains. However, treatment with ribavirin resulted in decreased specific infectivity only for MNV-3 treated with the highest concentration (400 μM). This suggests that the lower mutagenic activity exhibited by ribavirin (Figure 2) is responsible for this limited effect on specific infectivity. MNV-3 was more sensitive to favipiravir and ribavirin than MNV-1 (Figures 3 and 4), although this difference could not be associated with significant variations in the mutation frequency values between these strains during infections in treated or untreated cells (Figure 2). This different behaviour may be related to the observation that MNV-3 has lower fitness than MNV-1 (compare virus titre yields for untreated infections in Figures 1, 3 and 4) which can result in a greater sensitivity to mutagenesis, as previously reported for other viruses (Sierra et al., 2000; Pariente et al., 2001). Confirming this possibility, a tissue culture-adapted MNV-3 population (18 serial passages in cell culture) responded similarly to MNV-1 when treated with 400 μM ribavirin (data not shown).

**Figure 3—figure supplement 1.**
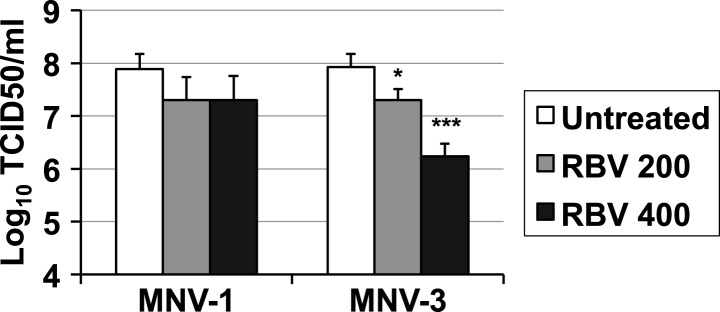
Reduced virus yields in ribavirin-mutagenised populations. RAW264.7 cells were infected with MNV-1 or MNV-3 recovered after 5 passages in the absence (untreated) or presence of ribavirin (200 or 400 μM) with the exception of 400 μM ribavirin-treated MNV-3 where passage 2 was used due to insufficient virus titres in subsequent passages. Infections were performed at an MOI of 0.01. After adsorption (1 hr at 37°C), virus inoculum was removed and complete media added to infected cultures. Infected cells were then incubated as mentioned in ‘Materials and methods’ for a total of 17 hr before freezing. **DOI:** http://dx.doi.org/10.7554/eLife.03679.008

**Figure 4—figure supplement 1.**
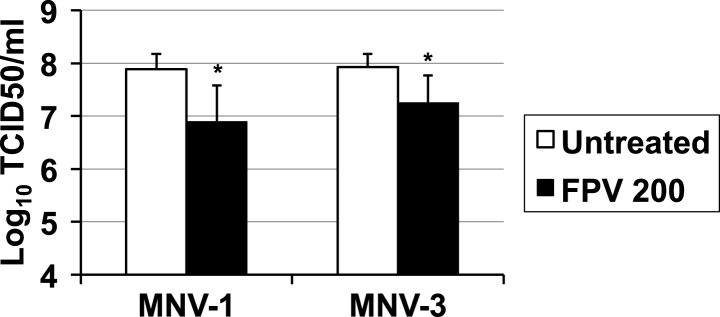
Reduced virus yields in favipiravir-mutagenised populations. RAW264.7 cells were infected with MNV-1 or MNV-3 recovered after 5 passages in the absence (untreated) or presence of favipiravir (200 μM) Infections were performed at an MOI of 0.01. After adsorption (1 hr at 37°C), virus inoculum was removed and complete media added to infected cultures. Infected cells were then incubated as mentioned in ‘Materials and methods’ for a total of 17 hr before freezing. **DOI:** http://dx.doi.org/10.7554/eLife.03679.010

To further investigate the effect of mutagenesis on virus fitness, RAW264.7 cells were infected with virus obtained after 5 passages in the presence of ribavirin or favipiravir (Figures 3 and 4) at the same MOI (0.01 TCID50/cell). Viral populations previously subjected to either favipiravir or ribavirin mutagenic treatment resulted in decreased virus titres, in agreement with a loss of infectivity as a consequence of mutagenesis (Figure 3—figure supplement 1, Figure 4—figure supplement 1).

### Favipiravir reduces the levels of infectious MNV in the faeces of persistently infected mice

**Figure 5.**
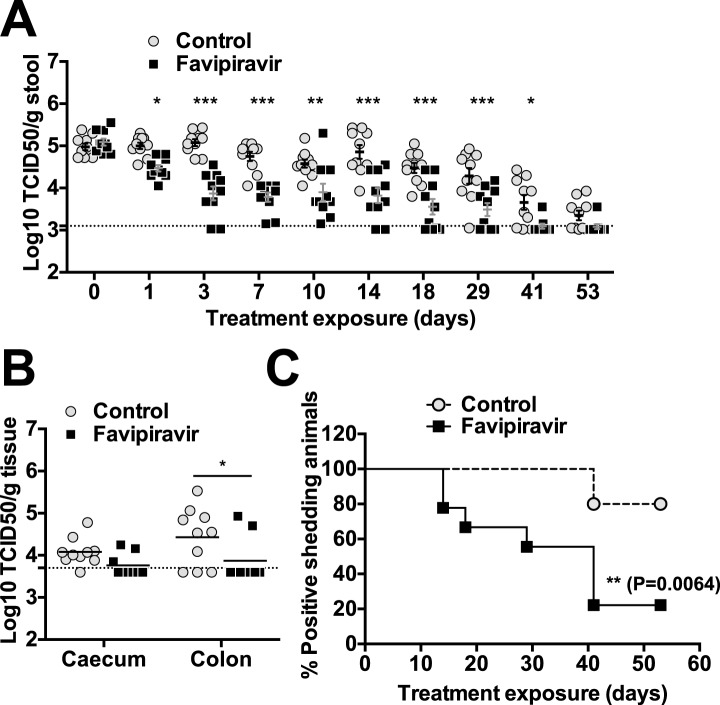
Favipiravir reduces infectious norovirus titres in mice faeces and tissues. Two groups of ten 4–5-week old C57BL/6 male mice were oral gavage-infected with 10^4^ TCID50 units of MNV-3. 4 weeks after virus inoculation, persistently infected animals were subjected to treatment with either 300 mg/kg animal of favipiravir twice a day (FPV) or with buffer (Control) for 8 weeks. From day 35 onwards, there are nine animals instead of 10 in the favipiravir-treated group (due to the accidental death of one mouse during dosing). (**A**) Virus titres in faeces of animals untreated or treated with favipiravir. Virus titres were determined by TCID50 assays of faecal samples supernatant previously resuspended at 100 mg/ml in PBS (p < 0.05, *; p < 0.01, **; p < 0.001, ***; 2-way ANOVA test). A dashed line indicates the limit of detection (3.02 Log10 TCID50/g stool). (**B**) Virus titres in caecum and colon of animals after 53 days of treatment with favipiravir. Virus titres were determined by TCID50 assays of homogenates of caecum and colon resuspended in DMEM at a concentration of 30 mg/ml (p < 0.05, *; 2-way ANOVA test). (**C**) Reduced positive shedding in animals treated with favipiravir. The percentage of animals shedding detectable virus titre along time, based on **A**, decreases faster in animals treated with favipiravir than in untreated animals (p = 0.0064, log-rank test). **DOI:** http://dx.doi.org/10.7554/eLife.03679.011

**Figure 6.**
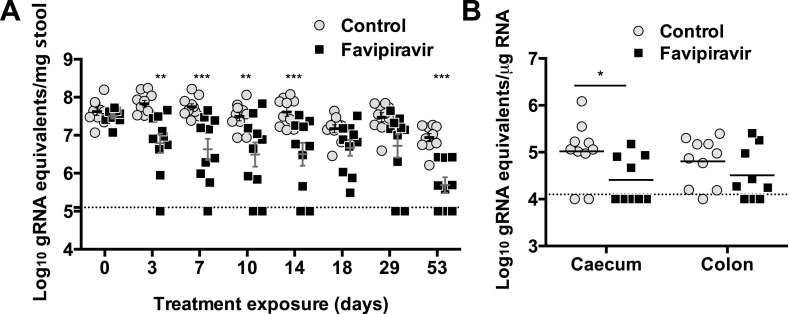
Long exposure to favipiravir results in significantly decreased norovirus RNA levels in animal tissues and faeces. Two groups of ten male C57BL/6 mice of 4–5-weeks were oral gavage-infected with 10^4^ TCID50 units of MNV-3. 4 weeks after virus inoculation, persistently infected animals were subjected to treatment with either 300 mg/kg animal of favipiravir twice a day (FPV) or with buffer (Control) for 8 weeks. At day 53, there are nine animals instead of ten in favipiravir-treated group due to the accidental death of one mouse during dosing. A dashed line indicates the limit of detection (10^2^ genome copy equivalent per mg of stool). (**A**) Viral genome copy equivalents isolated in faecal samples (p < 0.05, *; p < 0.01, **; p < 0.001, ***; 2-way ANOVA test). Viral RNA extracted was then reverse transcribed and quantitated as described in ‘Materials and methods’. (**B**) Viral genome copy equivalents isolated in caecum and colon (p < 0.05, *; 2-way ANOVA test). **DOI:** http://dx.doi.org/10.7554/eLife.03679.016

The efficacy of ribavirin and favipiravir as antiviral compounds in vivo was next investigated in C57BL/6 mice persistently infected with MNV-3. Animals were infected 4 weeks before the beginning of the treatment to allow the establishment of a persistent infection. In a preliminary experiment (Figure 5—figure supplement 1), we found that all the animals treated with favipiravir had lower virus titres (5/5) in their faeces while only 2 out of 5 animals treated with ribavirin had consistently lower titres (Figure 5—figure supplement 1). This suggests that favipiravir is more efficient than ribavirin in the control of norovirus replication in vivo, in agreement with data obtained in tissue culture. Hence, we conducted further experiments to determine whether favipiravir can drive lethal mutagenesis of MNV-3 during persistent infection in mice. With this aim, we treated mice persistently infected with MNV-3 with either placebo or 600 mg/kg/day of favipiravir for 8 weeks (Figure 5). Favipiravir was effective in the control of persistent norovirus replication in vivo with decreased virus titres and RNA levels in faeces observed (Figures 5 and 6) at very beginning of the treatment (day 1) and throughout the entire treatment period (day 53). A predicted half-life of 31 days is calculated for virus clearance in favipiravir-treated mice in contrast with 122 days in the untreated group (p = 0.0064, log-rank test; Figure 5B).

**Figure 5—figure supplement 1.**
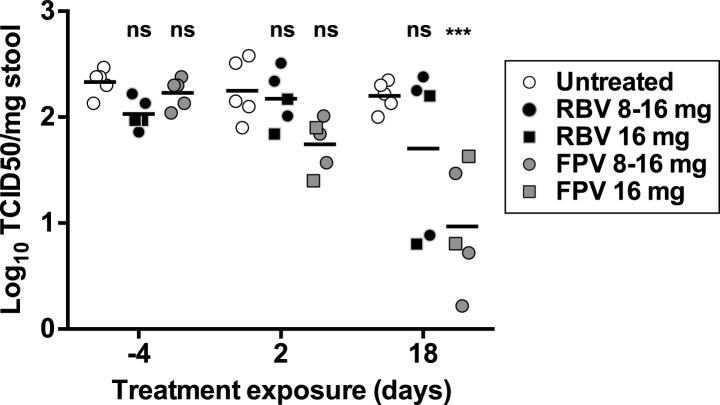
Antiviral activity in vivo of favipiravir and ribavirin. 4–5-week old male C57BL/6 mice were oral gavage-infected with 10^4^ TCID50 units of MNV-3. 4 weeks after infection, animals underwent daily treatment with mutagenic compounds for a total of 18 days (day 0 to day 17), with the exception of days 6 and 13 when the animals were not treated. Animals were dosed by oral gavage with 8 mg/day (3 animals) or 16 mg/day (2 animals) of either ribavirin or favipiravir for 12 days. Afterwards, the animals were treated with 16 mg/day of the same drug for six additional days. Virus titres obtained in faecal samples isolated 4 days before treatment began (−4) and after 2 and 18 days of treatment (***; p < 0.001, 2-way ANOVA test). **DOI:** http://dx.doi.org/10.7554/eLife.03679.012

**Figure 5—figure supplement 2.**
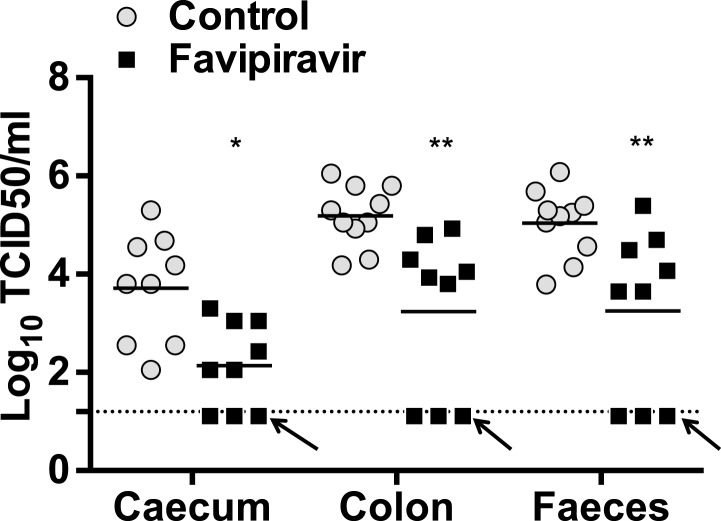
Infectious virus rebound after blind passage of faecal and tissue sample homogenates in RAW264.7 cells. Virus titres obtained after blind passage infection in RAW264.7 cells of faeces, caecum, and colon homogenates obtained from animals at treatment day 53 (Figure 5A,B). An arrow indicates samples from the same three animals that remained negative after blind passage, indicating extinction. These samples remained negative by TCID50 assay and qPCR after three consecutive blind passages. **DOI:** http://dx.doi.org/10.7554/eLife.03679.013

**Figure 5—figure supplement 3.**
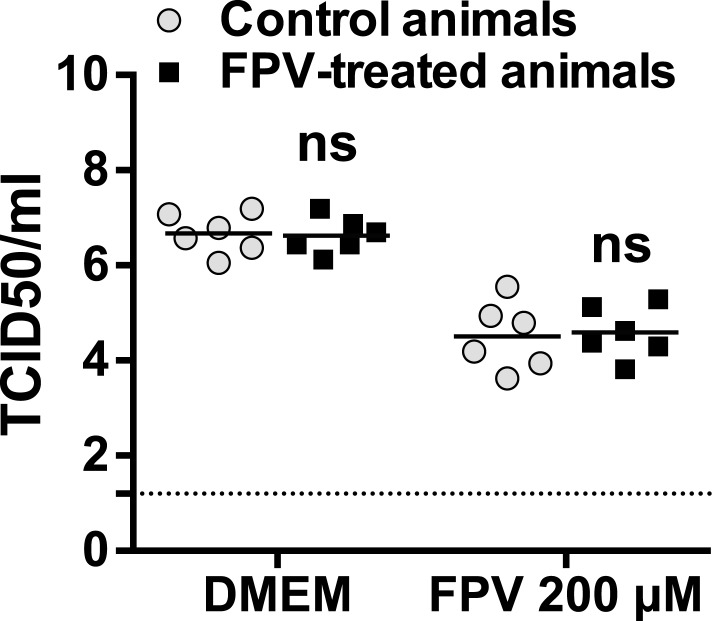
Sensitivity to favipiravir of virus isolated in animal faeces. Faecal virus samples obtained from untreated or treated mice during 53 days were amplified by blind passage in RAW264.7 cells and treated with favipiravir to examine the possible presence of resistance. **DOI:** http://dx.doi.org/10.7554/eLife.03679.014

**Figure 5—figure supplement 4.**
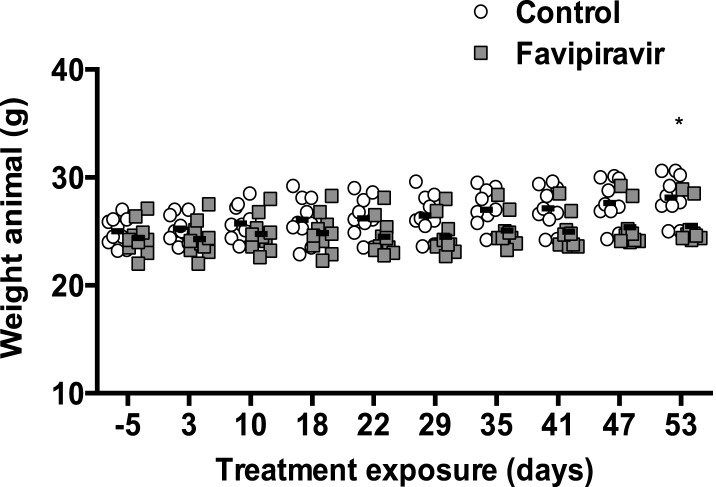
Reduced weigh increase in favipiravir-treated mice. They are represented animal weights before (−5) and during the treatment with placebo or favipiravir (*; p < 0.05; 2-way ANOVA test). **DOI:** http://dx.doi.org/10.7554/eLife.03679.015

**Figure 6—figure supplement 1.**
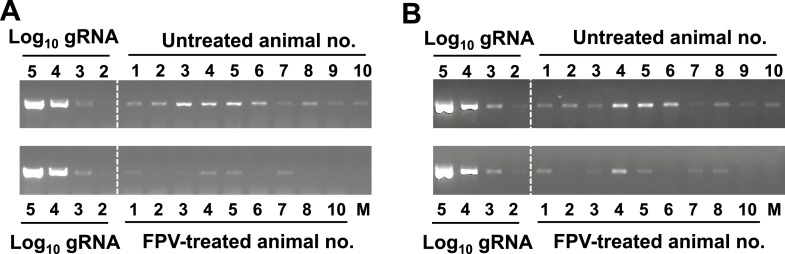
Semi-quantitative analysis of viral RNA in favipiravir-treated and control mice. Viral RNA was extracted and reverse transcribed as described in ‘Materials and methods’ (Arias et al., 2012a). Standard curve was obtained with known amounts of in vitro transcribed MNV-3 RNA (10^2^ to 10^5^ genome equivalents) that were PCR amplified after reverse transcription. (**A**) RT-PCR amplification of faecal samples isolated from animals treated during 53 days with favipiravir or placebo. RNA isolated in 50 μg of faeces was RT-PCR amplified and agarose-gel resolved as explained above. (**B**) RT-PCR amplification of RNA extracted from colon samples isolated from animals treated for 8 weeks with favipiravir or placebo. 50 ng of RNA isolated from different animal colon tissues were RT-PCR amplified as explained above. **DOI:** http://dx.doi.org/10.7554/eLife.03679.017

After 53 days of treatment with favipiravir, infectious virus titres were undetectable in the faeces of 7 out of 9 animals, while only 2 out of 10 animals had undetectable titres in the control group, suggesting that the infections were cleared as a result of favipiravir treatment (Figure 5A,C). Similar results were obtained when homogenates of caecum and colon collected at the end-point (day 53) were analysed for the presence of MNV (Figure 5B). To confirm whether these samples were negative, we carried out a blind passage in RAW264.7 cells. We confirmed that three treated animals were negative for all the samples analysed (faeces, caecum, and colon) while the remaining treated and untreated animals (6 and 10 respectively) were positive for MNV (Figure 5—figure supplement 2). All these three animal samples remained negative by titration and qPCR after 3 passages in the absence of favipiravir. These results suggest that favipiravir has assisted in clearing the infection in 33% of treated mice.

To investigate whether favipiravir treatment in vivo resulted in the selection of adapted MNV-3 variants, we carried out infections in RAW264.7 cells with virus samples recovered from mice. We did not identify differences in the sensitivity to favipiravir between samples isolated from treated and untreated animals, suggesting no adaptation to the treatment (Figure 5—figure supplement 3).

Quantification of MNV RNA in faecal samples also showed lower viral levels in treated mice than in control animals for the duration of the study (Figure 6). The same three animals that were negative for MNV-3 above showed viral RNA levels in faeces below the detection limit after 53 days of treatment, determined both by RT-qPCR (Figure 6), and RT-PCR followed by agarose gel analysis (Figure 6—figure supplement 1), while all placebo-treated animals showed high levels of viral RNA. Quantification of viral RNA extracted from caecum and colon, the major tissues for virus replication during persistent infections (Arias et al., 2012a), also confirmed that viral RNA could not be detected in these three treated mice that contain no amplifiable infectious virus in faeces and tissues, further supporting that virus infection was cleared in these animals (Figure 6).

### Favipiravir induces increased error frequencies in norovirus replication in vivo

**Figure 7.**
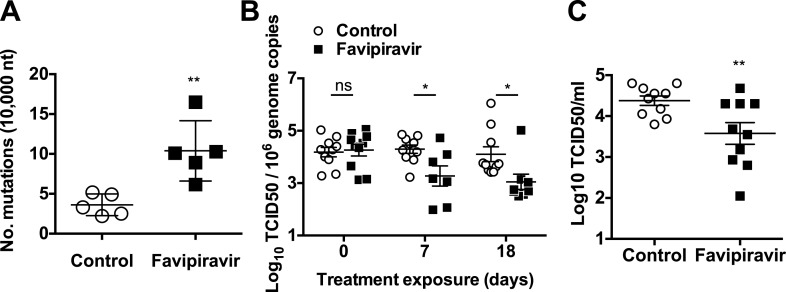
Increased mutation frequencies and decreased infectivity in virus populations isolated from favipiravir-treated animals. (**A**) Mutation frequency in virus isolated in faecal samples. Every value in the graph represents the virus mutation frequency in a different animal faecal sample after 18 days of treatment. Mutation frequencies are represented as the average number of nucleotide substitutions found in every 10,000 nucleotides sequenced. (0.001 < p < 0.05, **; *t* test). (**B**) Decreased infectivity in viral RNA isolated from favipiravir-treated animals. Viral RNA isolated from placebo and favipiravir-treated animal faecal samples was quantified and 2 × 10^5^ genome copy equivalents were lipofected in semiconfluent BHK-21 cell monolayers. At 24 hr post-transfection, cells were freeze-thawed and the resulting virus yields determined by TCID50 assays in RAW264.7 cells. They are represented as the virus titres obtained per 10^6^ genome copies isolated from the faeces of infected animals before treatment (day 0) and at treatment days 7 and 18. (**C**) MNV recovered from favipiravir-treated animals shows reduced replication yields. Virus isolated from animal faeces treated with favipiravir were first amplified in RAW264.7 cells allowing virus replication for 24 hr. Recovered viruses were titrated and 0.01 TCID50 units/cell applied to new RAW264.7 cell monolayers. Virus infections were collected at 8 hr post-infection and the cultures freeze-thawed to release infectious virus (**; p < 0.01; *t* test). **DOI:** http://dx.doi.org/10.7554/eLife.03679.018

To clarify if favipiravir antiviral activity observed in vivo was associated with mutagenesis, we examined the mutation frequency of viral populations shed in the faeces of five different animals in each group. The mutation frequencies found in virus samples from favipiravir-treated animals were greater than in placebo-treated mice with an average of a 2.9-fold increase relative to control animals being observed (Figure 7A). We also determined the mutation frequencies in three samples isolated from ribavirin-treated animals in the preliminary experiment, and they were similar to those observed for placebo-treated animals (4.0 ± 2.2 vs 3.6 ± 1.4 substitutions per 10,000 nucleotides sequenced, respectively). These results suggest that the antiviral activity observed for favipiravir in vivo is linked to mutagenesis, and the clearance of infection in some of these animals is the consequence of lethal mutagenesis of persistently replicating virus.

**Figure 7—figure supplement 1.**
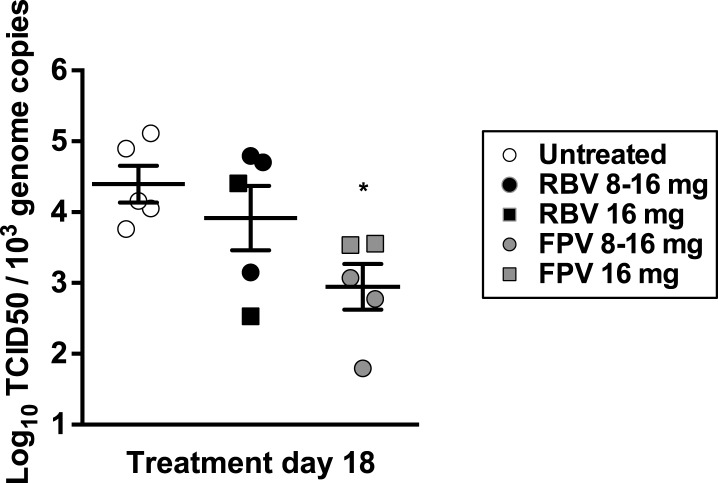
Specific infectivity of viral RNA isolated from treated animals. 4–5-week old male C57BL/6 mice were oral gavage-infected with 10^4^ TCID50 units of MNV-3. 4 weeks after infection, animals underwent daily treatment with mutagenic compounds during a total of 18 days (day 0 to day 17) with the exception of days 6 and 13 when the animals were not treated. Animals were dosed by oral gavage with 8 mg/day (3 animals) or 16 mg/day (2 animals) of either ribavirin or favipiravir for 12 days. Afterwards, the animals were treated with 16 mg/day of the same drug for six additional days. Viral RNA isolated from placebo-, ribavirin-, and favipiravir-treated animal faecal samples were quantified and 5 × 10^4^ genome copy equivalents lipofected in semiconfluent BHK-21 cell monolayers. At 24 hr post-transfection cells were freeze-thawed and resulting virus yields determined by TCID50 assays in RAW264.7 cells. They are represented the infectivity values for RNA samples isolated from faeces collected at day 18. **DOI:** http://dx.doi.org/10.7554/eLife.03679.019

### Decreased specific infectivity and fitness in favipiravir treated populations in vivo

To investigate whether favipiravir-induced mutagenesis in vivo resulted in decreased infectivity of viral populations, we firstly isolated and quantified viral RNA from faeces at various times during the treatment of animals with favipiravir and used an equivalent genome copy number to transfect BHK-21 cells (Figure 7B). BHK-21 cells support MNV replication but not virus (re)infection, resulting in a single round of replication for the transfected genomes and a better correlation between infectious genome units and virus titre. Thus, this approach provides an indirect measure of viral genome specific infectivity, as only viable genomes will result in the recovery of infectious virus. Viral titres recovered were consistently lower in samples obtained from favipiravir-treated animals than in placebo-treated animals (Figure 7B), suggesting decreased specific infectivity as a consequence of increased mutagenesis. Virus titres for genomes isolated from ribavirin-treated animals showed no significant difference with untreated animals (Figure 7—figure supplement 1).

To compare whether mutagenesis in treated animals resulted in lower fitness of virus samples recovered, we determined the relative replication rates of virus populations recovered from mice after 18 days of treatment. To this aim, we carried out MOI controlled infections (0.01 TCID50/cell) in RAW264.7 cells using virus previously isolated from faecal samples and propagated once in cell culture. Virus yields obtained using virus from favipiravir-treated animal samples were lower on average than those isolated from placebo-treated animal samples, suggesting that mutagenesis induced by favipiravir results in a viral fitness cost in vivo (Figure 7C). Thus, favipiravir causes increased mutagenesis and decreases the specific infectivity and fitness of norovirus in vivo which supports an antiviral activity mediated by enhanced mutagenesis.

## Discussion

Lethal mutagenesis has been the subject of numerous studies in cell culture in the last few years as an alternative approach to classical antiviral therapies (Perales et al., 2011). Due to the elevated mutation frequencies in RNA viruses, it was predicted that an increase in the replication error rate might result in the extinction of the viral population (Eigen, 2002; Domingo et al., 2010). However, several in vivo studies carried out up to date have resulted in insufficient evidence to support lethal mutagenesis as a practical approach at the clinical level. In this study, we show that favipiravir, a purine nucleoside analogue, can cause the extinction of an RNA virus during replication in its natural host. The antiviral activity observed in vivo is associated with increased mutation frequencies and, importantly, reduced infectivity in virus samples isolated from the treated animals. These are features typically observed in viral quasispecies approaching extinction during lethal mutagenesis which constitutes a proof-of-principle for this antiviral strategy.

Favipiravir was initially identified as an antiviral compound for the treatment of influenza virus infection (Furuta et al., 2002; Sidwell et al., 2007) and currently is being tested in a phase 3 clinical trial. Previous studies on mice and data from clinical trials in humans show that favipiravir is well tolerated in vivo with much less toxicity exhibited than ribavirin (Gowen et al., 2007). In this study, we observed no major side effects in the mice treated during the 8 weeks. The only apparent side-effect is that treated mice show a modest reduced rate of weight gain compared to the control group (Figure 5—figure supplement 4).

In addition to its activity in vivo against influenza virus and data shown here on MNV, favipiravir is efficient in the control and clearance of a broad number of RNA viruses including picornavirus, parmyxovirus, bunyavirus, arenavirus, and togavirus (Furuta et al., 2002; Gowen et al., 2007; Mendenhall et al., 2011; Caroline et al., 2014; Oestereich et al., 2014; Smither et al., 2014). Importantly, favipiravir has shown efficient antiviral activity in mouse models for Ebola virus infections, which has led some African countries to consider using this drug for the control of the current outbreak (Ikuomola, 2014). Further studies are needed to elucidate if the mechanism of action of favipiravir against these other viruses is lethal mutagenesis, and if it constitutes a possible universal antiviral mutagen for the clinical treatment of viral diseases. In particular, an attractive possibility would be to study the effect of favipiravir in the control of HCV, particularly given the lower toxicity displayed by favipiravir.

These results are also relevant for the development of antiviral strategies to control human norovirus (HuNoV) infections for which there are currently no licenced vaccines or antiviral therapies. Due to the absence of cell culture systems to recover and propagate infectious HuNoVs, MNV has been suggested as a potential surrogate system (Wobus et al., 2006). HuNoVs are a significant cause of non-bacterial gastroenteritis with large economic losses typically associated with frequent outbreaks in contained environments (>$160 million in UK hospitals alone). In addition, they have been linked to other important disorders such as ulcerative colitis and persistent diarrhoea (Murata et al., 2007; Ludwig et al., 2008; Khan et al., 2009; Capizzi et al., 2011; Bok and Green, 2012). Chronic norovirus infections constitute a major health problem in immunocompromised patients with no treatment yet available (Bok and Green, 2012). Favipiravir or other derivatives with improved pharmacokinetic properties may constitute an attractive candidate for the treatment of these patients. The rapid evolution and large mutation frequencies of norovirus replicating in immunocompromised patients (Bull et al., 2012) suggest that antiviral mutagenesis could be an effective approach. Favipiravir could also be considered as prophylactic treatment to reduce virus dissemination during the course of an outbreak, especially in contained environments such as hospitals or nurseries. The data obtained for persistently infected mice support this possibility with lower virus yields shed by treated animals since an early time during treatment (Figure 5).

Recent evidence suggests that the combination of a mutagenic compound with a classical antiviral molecule can be more efficient in the extinction of a virus than the use of the compound alone or the combination of classical inhibitors only (Pariente et al., 2001; Perales et al., 2009). Inhibitors with antiviral activity in vivo have been identified for multiple RNA viruses, including norovirus (Perry et al., 2012; Rocha-Pereira et al., 2013), encouraging further studies in this direction. Given its significant efficacy in the control of different RNA viruses, favipiravir or other derivatives with improved pharmacokinetics constitute attractive candidates to become universal antiviral compounds against viral diseases via lethal mutagenesis.

## Materials and methods

### Ethics

Studies with mice were performed in the Department of Pathology BSU Unit (PCD 80/2802) after ethical review by the University of Cambridge Review Panel and subsequent approval by the UK Home Office (PPL70/7689). All animal procedures and care conformed strictly to the UK Home Office Guidelines under The Animals (Scientific Procedures) Act 1986.

### Cells, infections, and reverse genetics recovery of viruses

Procedures for the cultivation of cells and MNV infections have been previously described (Arias et al., 2012a). Murine leukaemia macrophage cells RAW264.7 were used for the propagation and titration (TCID50 assay) of murine norovirus 1 and 3 (MNV-1 and MNV-3) used in this study. Baby hamster kidney cells (BHK-21) were used for the determination of infectivity in viral genomes isolated from animal samples. All the different cell lines were cultured in Dulbecco's modified Eagle medium (DMEM) with 10% FCS, 100 U/ml penicillin, and 100 mg/ml streptomycin (complete DMEM) and maintained at 37°C with 10% CO_2_. MNV-1 and MNV-3 strains used in this study were obtained after reverse genetics recovery of infectious virus as previously described (Arias et al., 2012b). Recovered viruses were then subjected to two consecutive passages in RAW264.7 cells. The resulting population was titrated and used as a passage 0 stock.

### Cell culture infections in the presence of mutagenic compounds

RAW264.7 cells were grown until they formed confluent monolayers (∼2 × 10^6^ cells in 35 mm diameter dish). Supernatant was then removed and replaced by 1 ml of complete DMEM containing either 200, 400, or 800 μM ribavirin or favipiravir and the cells were incubated for two additional hours at 37°C and 10% CO_2_. Cells were inoculated with 200 μl of virus at the multiplicity of infection (MOI) indicated and incubated for 1 hr at 37°C and 10% CO_2_. Supernatants were removed, cells washed to eliminate unattached virus, and 2 ml of fresh media containing ribavirin or favipiravir were added to each well. Cell cultures were collected at 24 hr post-infection and virus released through two consecutive freeze–thaw cycles.

For experiments involving serial passage of virus populations in the presence of favipiravir or ribavirin, passage 1 cells were infected at an MOI of 0.01 TCID50/cell with MNV-1 or MNV-3. In subsequent passages, 200 μl of neat virus from the previous passage (1/10 of total virus) were applied to a new monolayer of cells.

### Viral RNA extraction, RT-PCR amplification, quantitative PCR, and mutation frequency analysis of virus populations

Viral RNA was extracted from 100 μl of viral samples, either supernatant from lysed infected cultures or PBS-resuspended faeces from animals, using EconoSpin columns (Epoch, Missouri City, TX), following protocols provided by the manufacturer. Viral RNA was quantified using a two-step qPCR approach following protocols described previously (Arias et al., 2012a).

For the calculation of mutation frequency in any virus population, we have followed protocols previously described (Beaucourt et al., 2011). Briefly, 4 μl of purified RNA were reverse-transcribed in 20 μl final volume using SuperScript III (Roche, Switzerland) as indicated by the manufacturer. 3 μl of cDNA were then PCR amplified using high fidelity KOD polymerase (Toyobo) using primers spanning genomic positions 3734 to 3770 and 6074 to 6034 in MNV-1 and 3734 to 3770 and 5738 to 5711 in MNV-3. PCR products were purified with EconoSpin columns (Epoch) and directly ligated in plasmid PCR Blunt using Zero Blunt PCR cloning kit (Life Technologies, Carlsbad, CA). Positive *Escherichia coli* colonies were identified by PCR screening with primers flanking the vector-cloning site and GoTaq polymerase (Promega, Madison, WI). The resultant PCR products corresponding to individual MNV cDNA clones were sequenced and the mutation frequency in each population calculated.

### Animal infections and antiviral treatment

4–5-week old male C57BL/6 mice were orally infected with 10^4^ TCID50 units of MNV-3 as previously described (Arias et al., 2012a). After 4 weeks, persistently infected animals were subjected once or twice daily to oral gavage treatment with ribavirin, favipiravir, or placebo. Ribavirin was dissolved in PBS before inoculation into animals, while favipiravir was resuspended in 0.5% carboxyl methyl cellulose (CMC) in PBS. For the preliminary experiment (Figure 5—figure supplement 1), animals were treated once or twice a day with 8 mg of ribavirin or favipiravir (∼300 or 600 mg/kg animal/day) for 18 days. For the larger experiment (10 mice per group) (Figures 5 and 6), animals were treated with 300 mg/kg animal favipiravir twice a day (600 mg/kg animal/day) for 8 weeks. Control animals were treated with 0.5% CMC in PBS. Faecal samples were collected at different time points along the treatment period and the presence of infectious particles and viral RNA determined. Animals were sacrificed after the 8-week treatment period and caecum and colon tissues collected to analyse the presence of viral RNA.

To confirm the absence of infectious virus in those faecal and tissue samples that showed negative infectivity by TCID50 assays, 100 μl of samples homogenates were used to infect 10^5^ cells. Infections were collected at 24 hr and subjected to two freeze-thawing cycles to release virus. The resulting virus was analysed by TCID50 assays. Those samples that remained negative after blind passage amplification were subjected to two additional blind passages in RAW264.7 cells as explained above, and the absence of infectious MNV was confirmed by TCID50 assays and qPCR.

To determine whether virus replicating in vivo has acquired resistance to favipiravir, virus samples obtained from animal faeces at treatment day 53 were blind passaged in RAW264.7 cells as mentioned above. The amplified virus samples were applied to 10^5^ RAW264.7 cell monolayers at an MOI of 0.01, and infections were allowed to proceed for 48 hr in the absence (DMEM) or presence of 200 μM favipiravir.
