# Supplementary material for: Favipiravir elicits antiviral mutagenesis during virus replication in vivo
Source: eLife. 2014 Oct 21;3:e03679. doi: 10.7554/eLife.03679 (PMC4204012; doi:10.7554/eLife.03679)
Supplement: Related texts

# Article commentary: Decision letter

**DOI:** 10.7554/eLife.03679.020

**Affiliations:** University of Utah, United States

eLife posts the editorial decision letter and author response on a selection of the published articles (subject to the approval of the authors). An edited version of the letter sent to the authors after peer review is shown, indicating the substantive concerns or comments; minor concerns are not usually shown. Reviewers have the opportunity to discuss the decision before the letter is sent (see review process). Similarly, the author response typically shows only responses to the major concerns raised by the reviewers.

Thank you for sending your work entitled “A mutagenic nucleoside can drive virus extinction during replication in vivo” for consideration at *eLife.* Your article has been favorably evaluated by Tadatsugu Taniguchi (Senior editor) and 3 reviewers, one of whom is a member of our Board of Reviewing Editors.

The following individuals responsible for the peer review of your submission have agreed to reveal their identity: Wes Sundquist (Reviewing editor) and Raul Andino, one of the other two reviewers.

The Reviewing editor and the other reviewers discussed their comments before we reached this decision, and the Reviewing editor has assembled the following comments to help you prepare a revised submission.

This manuscript documents the increased efficiency of favipiravir over ribavirin in inducing lethal mutagenesis in RNA viral infection. The loss of infectivity and the increased genetic burden during passage in tissue culture and in mice is chronicled for murine norovirus. The persistent norovirus infection developed by this laboratory is an excellent system to study clearance of chronic viral infections.

Figures 1-4 show nice cell culture experiments in which mutagens ribavirin and favipiravir reduce and even extinguish virus, cause mutation accumulation in the viral RNA, and reduce the particle-to-PFU ratio in a way expected if the genetic burden on the viral genome increases. Although these kinds of experiments have been done previously in the Andino, Kirkegaard, Vignuzzi, and other laboratories, none have been done with favipiravir, which is more effective, less toxic and shows a different pattern of mutagenesis. This is a thorough and interesting study that presents the first clear experimental demonstration that this type of compound can virus disappearance by introduction of mutations into the viral genome.

The work will be an important contribution to the field once several issues have been addressed:

1) The authors have overstated their case and should “tone down” their rhetoric in several places. These include:

a) The claim that this is “the first proof of principle for lethal mutagenesis as a feasible therapeutic approach”. “Feasible” is a very subjective term. Many papers have shown inhibition of virus growth and pathogenesis in mouse models in the presence of ribavirin, and many papers have shown increased mutagenesis in viruses grown in cell culture. It is true that there are very few studies that have both shown increased mutation accumulation as well as treatment effectiveness in the same paper. Other examples of the in vivo effects of ribavirin on a reduction in viral titers and either an increase or an altered mutation spectrum in mice includes:

Chung et al., 2013, J. Virol. 87:10997.

Dietz et al., 2013, cited in paper. This paper shows a change in mutation profile in the presence of ribavirin in humans concomitant with a decrease in viral load.

b) The exaggerated claim in the title that the compound “can drive viral extinction during replication in vivo”. Figure 5 shows reduction of virus below the detection limit, but virus also 'extinguishes' in this experiment in the untreated mice, just later. Therefore, the mice were not actually 'chronically infected'.

c) Similarly, in Figure 6A, the authors show the disappearance of viral RNA from the faeces of 7/9 favipiravir-treated animals while this occurred in only 2/10 control animals. This is a trend but probably not significant and not sufficient to make it into the title. It is not clear in this case, also, that the mice in this experiment were 'chronically infected' because the amount of RNA was certainly trending downward even in the control.

2) The issue of “viral extinction” is an important one. For a virus to be driven to extinction, it should not recrudesce in the absence of drug. Have the authors done this experiment? (And if not, it should be done.)

3) The enhanced efficacy of ribavirin at early time points could either be “due to a different mechanism of action or an additional antiviral activity” or the different mutational spectrum could render it less possible for adaptive mutations to accumulate. This is the major argument against the concept of lethal mutagenesis, promoted by several laboratories including that of James Bull.

4) That the mutation rate increases in correlation with compound treatment does not mean that it was causal. Of course, it is likely that it was, but the authors should tone down their statements of causality. However, as the authors state on in the Introduction: “some studies have not found increased mutagenesis as a factor contributing to the associated antiviral activity”. Please parse out what is different, then, about the logic of the present study.

5) Figure 6. “Semi-quantitative PCR” does not contribute to the conclusions of the study and these panels should be deleted.

6) The favipiravir mechanism of action was suggested to direct inhibition of the RNA-dependent RNA polymerase, and the paper would be strengthened by establishing whether or not the antiviral effect is not in part a direct inhibition of RNA synthesis. Directly inhibition of RdRp could be tested by doing kinetics experiments in intact cells using RT-PCR because mutagenic effects should only be apparent after accumulation of mutations that manifest after few rounds of replication (particularly at high MOI), whereas direct effects on the RdRp may be clear on one step-replication curve. An alternative is to perform biochemical analyses of the effects of favipiravir on the purified polymerase.

7) Another important issue is whether or not the virus can develop resistance to favipiravir, and could also demonstrate that the central tenet of the study is correct. Have the authors done such experiments?

8) Figure 7C does not seem to provide any new information. Please clarify or delete.

9) Figure 1 A and B. The authors should provide the concentration of compound on the X axis of the graphs or provide a legend to make it clear. The figure should indicate the compound, concentration, and virus strain directly in the image.

10) Figure 1 C and D should be removed or clarified. These figures show that ribavirin inhibited norovirus replication at early time points, but that favipiravir gradually inhibited replication. The authors argue that this difference indicates that the two compounds act via different mechanism of actions. However, it is alternatively possible that the delayed inhibition shown by favipiravir compared to ribavirin reflects the fact that it takes several more steps to convert favipiravir to the active form compared to ribavirin. For example, favipiravir is predicted to be ribosylated and polyphosphorylated (Antimicrob Agents Chemother, 2005 Mar;49(3):981-6) whereas ribavirin only needs to be converted to the triphosphorylated form. Additional experiments would need to be performed to draw conclusions from this data.

11) Figure 2 A and B. A) What is the total number of mutations identified for each treatment group? B) Please supply the individual mutation numbers in a table format with raw numbers so that the reader can determine the actual numbers that the % represents.

12) “A signature feature for error catastrophe in a virus population subjected to mutagenesis is a decrease in virus infectivity.” The authors probably mean a decrease in specific infectivity since all antivirals would be expected to decrease virus infectivity.

13) The authors state that the vRNA levels from the colon and caecum are lower or undetectable in the treated mice, but this is only true for the caecum, not the colon.

# Reply: Author response

**DOI:** 10.7554/eLife.03679.021

*1) The authors have overstated their case and should “tone down” their rhetoric in several places*. *These include:*

a) The claim that this is “the first proof of principle for lethal mutagenesis as a feasible therapeutic approach”. “Feasible” is a very subjective term. Many papers have shown inhibition of virus growth and pathogenesis in mouse models in the presence of ribavirin, and many papers have shown increased mutagenesis in viruses grown in cell culture. It is true that there are very few studies that have both shown increased mutation accumulation as well as treatment effectiveness in the same paper. Other examples of the in vivo effects of ribavirin on a reduction in viral titers and either an increase or an altered mutation spectrum in mice includes:

*Chung et al., 2013**, J. Virol. 87:10997*.

*Dietz et al., 2013**, cited in paper. This paper shows a change in mutation profile in the presence of ribavirin in humans concomitant with a decrease in viral load*.

In hindsight, we agree with the reviewers in this point. As they state, others have provided evidence to suggest that ribavirin could be causing lethal mutagenesis in vivo. We have edited the manuscript to tone down the rhetoric and make the text more accurate. We have also modified the title of the paper as suggested below to: ‘Favipiravir elicits antiviral mutagenesis during virus replication in vivo’

We have also modified the text to highlight the advances made in the present manuscript in the field of lethal mutagenesis. Specifically, our data support that lethal mutagenesis occurs in vivo since increased mutagenesis is associated with decreased specific infectivity and fitness of the virus population. We have now modified the text accordingly.

*b) The exaggerated claim in the title that the compound “can drive viral extinction during replication in vivo”*.

We agree with the reviewers that our title might be exceeding the evidence obtained. Thus, we have changed the title to more clearly reflect our data.

*Figure 5* *shows reduction of virus below the detection limit, but virus also 'extinguishes' in this experiment in the untreated mice, just later. Therefore, the mice were not actually 'chronically infected'*.

MNV establishes a persistent infection that can be typically tracked by PCR for at least 8 months (McFadden et al 2013, Nucleic Acids Res) confirming the establishment of long-term persistent infections. We have included a an additional graph in Figure 5 (panel C) showing the proportion of positive shedding animals along time to better show differences between treated and untreated groups. From this graph, we have extrapolated the expected half-life for virus clearance: ∼120 days in the untreated group and 31 days in the favipiravir group (P=0.0064).

We have also now included data of animal samples that have been blind passaged in RAW264.7 cells (further explained below). Virus rebound was observed in all untreated animals while 3 out of 9 favipiravir-treated animals remained negative (included as a supplementary figure). We have modified the text now to include this information.

We have also modified Figure 5 (now Figure 5A) to make the data simpler. In particular, we have removed data points for days -12 and -5 as they are superfluous to the day 0 data. We have also included panel B with virus titres in caecum and colon of animals after 53 days of treatment.

*c) Similarly, in* *Figure 6A**, the authors show the disappearance of viral RNA from the faeces of 7/9 favipiravir-treated animals while this occurred in only 2/10 control animals. This is a trend but probably not significant and not sufficient to make it into the title. It is not clear in this case, also, that the mice in this experiment were 'chronically infected' because the amount of RNA was certainly trending downward even in the control*.

We have assumed that the reviewers here refer to Figure 5 and not to Figure 6A. We have modified Figure 5 as explained above. Although virus titres are substantially reduced along time in the control group, viral RNA levels are maintained relatively stable with a modest reduction between day 0 and day 53 (4.6-log versus 3.9-log mean values). All the animals in the control group are positive while 3/9 animals in the favipiravir-treated group show undetectable RNA levels in faeces and tissues. As described above, the Figure 5 and text have been modified to clarify this.

*2) The issue of “viral extinction” is an important one. For a virus to be driven to extinction, it should not recrudesce in the absence of drug. Have the authors done this experiment? (And if not, it should be done*.*)*

We have performed additional experiments and confirmed that extinction occurs in cell culture for MNV-3 treated with 400μM favipiravir (Figure 4). Virus passage 5 subjected to three blind passages in the absence of drug in RAW264.7 cells did not recover any infectious virus by qPCR or TCID50 assay.

We are unable to determine whether a rebound in viral infection occurs in the absence of favipiravir in vivo, as we sacrificed the animals at day 53 (end-point). To determine whether virus infection has been cleared in favipiravir-treated animals, we have blind passaged samples isolated from faeces and tissues collected post-mortem (day 53) in RAW264.7 cells. No virus rebound was observed in any faecal or tissue sample from 3 treated animals (33% of treated animals) after 3 blind passages cell culture, which suggests that virus extinction has occurred in these animals. This new data are included as Figure 5–figure supplement 2.

We have now included the data of blind passages of animal samples in the manuscript.

*3) The enhanced efficacy of ribavirin at early time points could either be “due to a different mechanism of action or an additional antiviral activity” or the different mutational spectrum could render it less possible for adaptive mutations to accumulate. This is the major argument against the concept of lethal mutagenesis, promoted by several laboratories including that of James Bull*.

We have now modified the text to include this possible alternative activity.

*4) That the mutation rate increases in correlation with compound treatment does not mean that it was causal. Of course, it is likely that it was, but the authors should tone down their statements of causality. However, as the authors state on in the Introduction: “some studies have not found increased mutagenesis as a factor contributing to the associated antiviral activity”. Please parse out what is different, then, about the logic of the present study*.

In this paper we have demonstrated that increased mutagenesis in vivo is associated with decreased specific infectivity of viral RNA isolated (Figure 7B, and in Figure 7–figure supplement 1), and also decreased replication fitness of virus samples isolated from faeces (Figure 7C), in experiments carried out in tissue culture in the absence of any drug. These two are main features of virus approaching extinction in tissue culture. Thus, our results suggest that virus replicating in favipiravir-treated animals are losing fitness and infectivity as a consequence of mutagenesis. We believe that this evidence constitutes a step beyond the majority of studies done in vivo to date and permit us to propose mutagenesis as a major antiviral trait associated to favipiravir treatment. We have now emphasised this link between mutagenesis and decreased fitness and specific infectivity in the text.

*5)* *Figure 6**. “Semi-quantitative PCR” does not contribute to the conclusions of the study and these panels should be deleted*.

We agree with the reviewer and we have now transferred these data to a supplementary figure (Figure 6–figure supplement 1). We consider that semi-quantitative PCR adds additional information to discriminate between negative and positive samples with values near the limit of detection by qPCR. However, we agree with the referees that might be redundant in the main text and we have now moved this figure to the supplementary information section.

*6) The favipiravir mechanism of action was suggested to direct inhibition of the RNA-dependent RNA polymerase, and the paper would be strengthened by establishing whether or not the antiviral effect is not in part a direct inhibition of RNA synthesis. Directly inhibition of RdRp could be tested by doing kinetics experiments in intact cells using RT-PCR because mutagenic effects should only be apparent after accumulation of mutations that manifest after few rounds of replication (particularly at high MOI), whereas direct effects on the RdRp may be clear on one step-replication curve. An alternative is to perform biochemical analyses of the effects of favipiravir on the purified polymerase*.

Our time-course data of high MOI-infected cells shows similar infectious virus production kinetics during the exponential phase of replication in the absence and presence of favipiravir, suggesting no effect of favipiravir on RNA replication. We have further investigated the effect of favipiravir and ribavirin on MNV RNA levels. We have obtained similar results to those obtained in kinetics of infectious virus, which further supports that favipiravir does not affect the exponential phase of MNV RNA replication (data not shown). We have now modified the text to include this information.

7) Another important issue is whether or not the virus can develop resistance to favipiravir, and could also demonstrate that the central tenet of the study is correct. Have the authors done such experiments?

We have not directly investigated whether MNV can adapt to replicate in the presence of favipiravir in tissue culture. The sequence analysis of MNV-1 and MNV-3 subjected to 5 passages in the presence of 200μM favipiravir did not identify any adaptive mutation in the virus polymerase.

Likewise, no signature changes have been found in the polymerase genes of populations isolated from treated animals (data not shown). To determine whether treatment with favipiravir selects for adaptive mutations, we have carried out an additional experiment to show if virus isolated from treated animals at treatment day 53 are less sensitive to favipiravir. However, the data obtained suggests that virus isolated from treated animals remains sensitive to favipiravir, with no difference observed between treated and untreated animal virus samples. This information is now explained in the text, and included as a new supplementary figure.

*8)* *Figure 7C* *does not seem to provide any new information. Please clarify or delete*.

Figure 7B and 7C are determinations of viral RNA infectivity and virus fitness, respectively. Thus, they show different complementary data. In Figure 7B we have directly transfected an equal amount of viral RNA extracted from faeces while in Figure 7C we have propagated infectious virus from faeces and carried out infections at same MOI. Figure 7B gives us a measure of specific infectivity as we are potentially transfecting both viable and non-viable viral RNA genomes into cells. Figure 7C identifies differences in fitness as we are infecting with an equal amount of infectious virus. We have now modified the text to make it clearer for the readers.

*9)* *Figure 1 A and B**. The authors should provide the concentration of compound on the X axis of the graphs or provide a legend to make it clear. The figure should indicate the compound, concentration, and virus strain directly in the image*.

We have modified the figure as suggested by the referees.

*10)* *Figure 1 C and D* *should be removed or clarified. These figures show that ribavirin inhibited norovirus replication at early time points, but that favipiravir gradually inhibited replication. The authors argue that this difference indicates that the two compounds act via different mechanism of actions. However, it is alternatively possible that the delayed inhibition shown by favipiravir compared to ribavirin reflects the fact that it takes several more steps to convert favipiravir to the active form compared to ribavirin. For example, favipiravir is predicted to be ribosylated and polyphosphorylated (Antimicrob Agents Chemother, 2005 Mar;49(3):981-6) whereas ribavirin only needs to be converted to the triphosphorylated form. Additional experiments would need to be performed to draw conclusions from this data*.

As mentioned above, we have carried out experiments with extended pre-incubation times (∼15 hours). Longer pre-incubation times with favipiravir do not substantially alter the observation made previously, suggesting that favipiravir do not inhibit MNV RNA synthesis. Further experiments to determine the kinetics of viral RNA synthesis in the presence of these compounds have correlated well with infectious virus kinetics, with no difference observed between untreated and favipiravir-treated cells at the exponential phase of replication. These new results have been included in the text.

*11)* *Figure 2 A and B**. A) What is the total number of mutations identified for each treatment group? B) Please supply the individual mutation numbers in a table format with raw numbers so that the reader can determine the actual numbers that the % represents*.

We have modified Figure 2, and included Table 1 with this information, as requested by the referees.

*12) “A signature feature for error catastrophe in a virus population subjected to mutagenesis is a decrease in virus infectivity.” The authors probably mean a decrease in specific infectivity since all antivirals would be expected to decrease virus infectivity*.

We have amended this sentence in the text.

*13) The authors state that the vRNA levels from the colon and caecum are lower or undetectable in the treated mice, but this is only true for the caecum, not the colon*.

We have reworded this part to make it clearer now. RNA levels are significantly lower in caecum. In colon are also lower but not significant.
